# Effects of rising temperatures and [CO_2_] on the physiology of tropical forest trees

**DOI:** 10.1098/rstb.2007.0032

**Published:** 2008-02-11

**Authors:** Jon Lloyd, Graham D Farquhar

**Affiliations:** 1 Earth and Biosphere Institute, School of Geography, University of LeedsLeeds LS2 9JT, UK; 2 Environmental Biology Group, Research School of Biological Sciences, Australian National UniversityCanberra, Australian Capital Territory 0200, Australia

**Keywords:** review, photosynthesis, climate change, plant growth

## Abstract

Using a mixture of observations and climate model outputs and a simple parametrization of leaf-level photosynthesis incorporating known temperature sensitivities, we find no evidence for tropical forests currently existing ‘dangerously close’ to their optimum temperature range. Our model suggests that although reductions in photosynthetic rate at leaf temperatures (*T*_L_) above 30°C may occur, these are almost entirely accountable for in terms of reductions in stomatal conductance in response to higher leaf-to-air vapour pressure deficits *D*. This is as opposed to direct effects of *T*_L_ on photosynthetic metabolism. We also find that increases in photosynthetic rates associated with increases in ambient [CO_2_] over forthcoming decades should more than offset any decline in photosynthetic productivity due to higher *D* or *T*_L_ or increased autotrophic respiration rates as a consequence of higher tissue temperatures. We also find little direct evidence that tropical forests should not be able to respond to increases in [CO_2_] and argue that the magnitude and pattern of increases in forest dynamics across Amazonia observed over the last few decades are consistent with a [CO_2_]-induced stimulation of tree growth.

## 1. Introduction

In an effort to guide thought as to how tropical forests may respond to climate change, there have been several reviews over the last few years, including Chambers & Silver (2004), Clark (2004) and Wright (2005), which have concluded that CO_2_ is unlikely to have any positive effect on forest productivity. But with other projected changes in the global climate system, especially increasing temperatures, almost certainly to result in some form of tropical forest decline, it seems to us that nearly all these reviews are, at best, conceptually inconsistent. For example, Clark (2004) argues that increasing atmospheric [CO_2_] should result in ‘little or no enhancement of biomass production rates’, which is equivalent to stating that the growth of tropical forests is currently not carbon limited. Yet, she also cites numerous examples of how higher temperatures might reduce tropical forest productivity through declined rates of net CO_2_ assimilation or enhanced rates of respiration, which is, of course, equivalent to assuming the exact opposite. Similarly, when discussing CO_2_, Wright (2005) suggests that ‘current photosynthesis levels meet, or even exceed, the carbon requirements for maintenance and growth’ but when discussing light availability concludes that ‘solar irradiance limits net primary production by closed-canopy forests…because shade limits photosynthetic carbon uptake by most leaves’. Such inconsistencies suggest a need for a coherent and objective framework in which to assess the probable effects of rising temperatures and atmospheric [CO_2_] on the physiology and growth of forest trees and this is the objective of this paper.

## 2. Photosynthesis, respiration and plant growth

We start with the simple question: ‘is the growth of tropical trees carbon limited?’, noting the relationship between photosynthesis and growth can be simply expressed as

(2.1) $$N_{\text{P}}=G_{\text{P}}(1-\varphi ),$$

where *N*_P_ is the rate of net primary production (new growth); *G*_P_ is the average rate of photosynthesis; and *φ* is the proportion of assimilated carbon lost through respiration of all organs (including the leaves at night) as well as through other processes such as volatile organic carbon emission (Harley *et al*. 2004) or exudation of organic acids and other carbohydrates from roots to the soil solution (Jones *et al*. 2003). From equation (2.1), we can reasonably infer that if *N*_P_ varies with a positive correlation to environmental factors known to stimulate *G*_P_ then positive evidence would be obtained that the growth of tropical forest trees is currently carbon limited.

It is extremely difficult to change ambient [CO_2_] for forest trees in a long-term experimental setting but a second strong environmental driver influencing tropical forest *G*_P_ is photon irradiance *Q*. Clarke & Clarke (1994) and Graham *et al*. (2003) showed that the *N*_P_ of neotropical trees responds positively to increases in *Q* on a seasonal or interannual basis. Keeling (2007) used a calibrated crown illumination index to show that tropical tree stem growth rates are positively correlated with tree canopy light exposure. Shading experiments show that the growth of young seedlings and saplings within tropical forests is almost always limited by light (Turner 2001). As far as we know, no mechanism other than a stimulation of photosynthesis by increased *Q* has been suggested to account for this. For understorey plants in tropical forests, [CO_2_] also has a strong stimulatory effect on plant growth (Würth *et al*. 1998), as would be expected from the strong [CO_2_] dependence of quantum yield (Ehleringer & Björkman 1977) combined with the observation of shaded tropical forest seedlings often only just surviving very close to their growth compensation points (Turner 2001). Thus, the answer to our question seems to be ‘yes’ and a reasonable place to start any analysis of effects of temperature on forest productivity is thus through effects on photosynthesis and respiration—after which we consider direct long-term effects of [CO_2_].

## 3. Temperature and photosynthesis

As shown in figure 1, temperature can affect photosynthesis through modulation of the rates of activity of photosynthetic enzymes and the electron transport chain (Sage & Kubien 2007) and, in a more indirect manner, through leaf temperatures defining the magnitude of the leaf-to-air vapour pressure difference *D*, a key factor influencing stomatal conductances. These two processes are termed here ‘direct’ and ‘indirect’.

### (a) Direct (mesophyll) effects

Direct temperature effects on photosynthetic metabolism involve changes in the activity of ribulose-1,5-carboxylase/oxygenase (Rubisco—the main carboxylating enzyme of photosynthesis) as well as processes associated with the regeneration of Rubisco's substrate, ribulose-1,5-bisphosphate (RuBP) through the Calvin cycle.

Temperature effects on Rubisco kinetics are complex with activation energies and Michaelis–Menten constants being affected (von Caemmerer 2000), but these temperature sensitivities are now reasonably well established (Bernacchi *et al*. 2001) and with an only modest sensitivity of RuBP carboxylation capacity to temperature (Sage & Kubien 2007; see figure 3). This temperature sensitivity varies little with genotype or growth conditions, although there may have been some genetic adaptation of Rubisco specificity to different levels of aridity (Galmes *et al*. 2005).

The maximum rate of RuBP regeneration, usually considered to be limited by the maximum rate of electron transport *J*_max_, is generally more sensitive to temperature than RuBP carboxylation capacity with its temperature sensitivity also varying substantially with growth conditions and/or genotype (June *et al*. 2004). Typical response curves for *J*_max_ are shown in figure 2, with data from a modelling study of ecosystem flux data from forest near Manaus (Mercado *et al*. 2006), leaf-level measurements from the same tower (Tribuzy 2005) and from soya bean leaves in the laboratory (June *et al*. 2004). The latter also showed that inhibition of *J*_max_ at supraoptimal *T*_L_ is fully reversible. Although the mechanism by which this reversibility occurs is unknown, the decline in *J*_max_ at high *T*_L_ is associated with an increase in the cyclic flow of electrons around photosystem (PS) I possibly serving as an important mechanism for the protection of both PS II and lipid membranes under high-temperature conditions (Sharkey & Schrader 2006). The considerable variation in the temperature sensitivity of *J*_max_ with both species and growth conditions (June *et al*. 2004) contrasts with the relatively constant temperature sensitivity of RuBP carboxylation/oxygenation. One general ‘rule of thumb’ then is that enzyme-mediated processes tend to be invariant in their temperature responses, but that, due to potential changes in fluidity and lipid composition (Sung *et al*. 2003), membrane-mediated processes may exhibit a considerable flexibility in temperature sensitivity according to growth conditions and genotype.

Although at lower [CO_2_] a reduction in the activity of the membrane-bound Rubisco activase at high *T*_L_ may also limit photosynthesis (Sage & Kubien 2007), where reductions in enzyme activity occur they are usually irreversible and associated with enzyme denaturation at *T*_L_>45°C. At such temperatures, irreversible destruction of the thylakoids may also occur, though the temperature at which this occurs depends upon the temperature at which leaves have developed (Berry & Björkman 1980).

### (b) Indirect (stomatal) effects

As evaporative demand *D* increases, stomata tend to close to reduce the rate of water loss through transpiration. Associated with this stomatal closure is a reduction in CO_2_ assimilation rate *A* due to a reduction in the rate of supply of CO_2_ to the chloroplast (Farquhar & Sharkey 1982). For rainforest environments, the absolute humidity of the air tends to remain more or less constant over a day (e.g. Shuttleworth *et al*. 1985) and so it is diurnal fluctuations in *T*_L_ that drive the variations in *D*. This changing *D* as *T*_L_ varies over the day gives rise to an apparent temperature dependence of *A* that is actually associated with stomatal responses to variations in *D* (Koch *et al*. 1994). This is the indirect temperature response in figure 1.

## 4. Tropical forest photosynthetic response to climate change

To quantify the importance of the above, we have developed a simple model of leaf-level photosynthesis, described in full in the electronic supplementary material. In brief, the model consists of standard equations of photosynthesis (Farquhar *et al*. 1980) interfaced with a hybrid of the stomatal models of Jarvis & Davies (1998) and Buckley *et al*. (2003), includes a surface energy balance, and is run for 2000 and 2040 using observational and model output for the region of Manaus in central Amazonia. For 2000, it is run for a current day [CO_2_] of 380 μmol mol^−1^. For 2040, it is run at [CO_2_] of 380 and 550 μmol mol^−1^. The latter is considered a likely [CO_2_] to be occurring in 2040. The difference between model predictions for 2040 and 2000 with [CO_2_]=380 μmol mol^−1^ gives an indication of the direct effect of predicted climate change on *A*. The difference between 380 and 550 μmol mol^−1^ in 2040 illustrates the extent to which climate change effects on *A* will be modified by increases in [CO_2_]. For all three [CO_2_]/climate assumptions, the indirect temperature effect is quantified by comparing model predictions with *g* always set to its maximum value, *G*=0.6 mol m^−2^ s^−1^ (see electronic supplementary material), with predictions applying equation (E7), which allow stomata to respond to changes in *D*.

Using supply and demand functions (Farquhar & Sharkey 1982), figure 3 shows *A* as a function of intercellular/chloroplastic [CO_2_], *C*, for *Q*=1500 μmol m^−2^ s^−1^. The different demand functions represent the direct temperature effects on *A*. The indirect (stomatal) supply functions are shown for both *g*=*G* and for *g* at the maximum *D* occurring in the simulations. Over a wide range of *T*_L_, the direct temperature effect is relatively small, with indirect effects, those being associated with reductions in *C* as *g* declines in response to increasing *D*, being much more significant.

The modelled annual rates of CO_2_ fixation (*G*_P_ in equation (2.1)), and maximum simulated *T*_L_ and *D* are shown in table 1. The difference in *G*_P_ between *g*≡*G* and *g* from equation (E5) for the 2000 climate (288 versus 207 mol C m^−2^ a^−1^) suggests an indirect temperature effect currently reducing *G*_P_ by approximately 30%. Maximum *T*_L_ and *D* are also much greater when *g* is allowed to vary (37.9 versus 34.2°C and 33.0 versus 20.8 mmol mol^−1^, respectively) due to higher sensible heat fluxes associated with stomatal closure at high *D*.

Comparing *G*_P_ at [CO_2_]=380 μmol mol^−1^ and *g*≡*G* for 2000 and 2400 suggests a direct temperature effect of climate change of less than 2% (295 versus 288 mol C m^−2^ a^−1^) with the slight increase attributable to higher *Q* in 2040. However, when stomatal interactions with the environment are included, *G*_P_ is reduced from 207 to 187 mol C m^−2^ a^−1^. This 10% reduction is due to higher *T*_L_ and *D* under the 2040 climate. Nevertheless, once higher [CO_2_] in 2040 is also taken into account, the indirect temperature effect reduction in *G*_P_ is more than negated by the increased availability of CO_2_. Similar results have also been reported for a fully coupled simulation in a global circulation model (Bounoua *et al*. 1999) and changes in *g* in direct response to higher [CO_2_] for our model are considered in the electronic supplementary material.

We conclude that temperature rises of the order of 1.5°C in Amazonia over the next 35 years or so are unlikely to have a significant direct effect on *G*_P_. Lower *g* due to higher *D* may reduce *G*_P_ below the value that would otherwise occur, but this effect will be more than offset by higher [CO_2_]. It is also important to recognize that current day indirect responses to temperature may bear little relationship to indirect effects of higher temperatures in the future. This is because increased temperatures associated with climate change will be accompanied by increases in sea temperatures, and therefore transiently increased evaporation from the oceans. Thus, on average, higher ambient humidities will occur and changes in *D* as global temperatures increase will be smaller than currently observed as temperatures vary on a daily or seasonal basis. Nevertheless, this effect may be offset by large-scale variations in precipitation patterns. Indeed, we have used 2040 for the simulation of future climatic effects on *G*_P_, because the Hadley Centre model used predicts significantly more rapid drying in the Amazon than most other general circulation models (GCMs), also with greater reductions in atmospheric humidity (Li *et al*. 2006). This leads to biome shifts after this time. Most other GCMs should therefore predict less severe increases in *D* than is the case here, and an even greater stimulation of *G*_P_ in 2040. Nevertheless, many of these precipitation estimates might also be substantially modified once detailed land-use change effects are taken into account (Moore *et al*. 2007).

## 5. Plant respiration and temperature

Expressed as a proportion of *G*_P_, plant respiration is *φ* in equation (2.1). It has long been known that for tropical forests *φ* tends to be higher than other ecosystems, typically ranging from 0.60 to 0.85 (Lloyd & Farquhar 1996). The reasons for this are unclear as available evidence suggests that, due to long-term acclimation, plants growing in warmer ecosystems should not necessarily have higher *φ* than their cooler counterparts (Atkin *et al*. 2005). One explanation, also consistent with a high proportion of tropical forest autotrophic respiration being below ground, is that tropical trees, growing on relatively infertile soils, need to invest a high proportion of their acquired carbon in the acquisition of phosphorus through mychorrizal associations and via high rates of organic acid exudation (Lloyd *et al*. 2001). The suggestion of Chambers & Silver (2004) that much of this tropical tree respiration is simply ‘wasteful’ is without foundation.

Consistent with the idea that the growth of tropical trees may be carbon limited, enhanced respiration losses have been invoked as one explanation for tropical tree growth reductions associated with longer-term warming trends (Clark 2007; Feeley *et al*. 2007), even though this is also at odds with the high levels of carbohydrate reserves generally found in tropical trees (Würth *et al*. 2005) indicating that carbohydrate availability is not limiting for growth (Wright 2005). But, in any case, the extreme apparent sensitivity of growth to temperature suggested by Clark (2007) and Feeley *et al*. (2007) requires a tropical tree respiration *Q*_10_>5 (see electronic supplementary material). This is in clear contradiction to observation (Meir *et al*. 2008) and other/additional explanations may exist. For example, the indirect detrimental effects of high temperatures on *A* demonstrated above may be linked to the growth reductions of tropical trees observed in warmer and drier years.

In the longer term, there is no reason to believe that tropical trees should not be able to acclimate their respiration to increasing temperatures (Atkin *et al*. 2005) and, even if enhanced respiratory losses do occur in the future, they should be more than offset by the capability for increased *G*_P_ as [CO_2_] increases simultaneously (table 1 and electronic supplementary material).

## 6. Other temperature-related factors

Although we have focused on photosynthesis and respiration, other physiological processes may also be important. For example, reproductive processes such as flowering and fruit set may be especially sensitive to high temperatures (e.g. Sato *et al*. 2006). Another process that may become increasingly important as tropical forests warm may be the ability of plants to emit isoprene, a process thought to help maintain membrane stability under moderately high temperatures (Sharkey & Schrader 2006).

## 7. Increasing [CO_2_] and plant growth

Although an increased [CO_2_] accompanying climate change should more than offset any detrimental effects of higher temperatures and increased *D* on tropical forest productivity, this does not necessarily mean that increasing [CO_2_] should also be serving to stimulate growth rates above those which would otherwise occur. Indeed, although increasing [CO_2_] provides a simple explanation for observed increases in recruitment and growth rates of Amazon forests over the last few decades (Lewis *et al*. 2004), this also being quantitatively consistent with theoretical predictions (Lloyd & Farquhar 1996), it has also been argued that a direct stimulation of plant productivity by CO_2_ cannot account for these growth responses observed (Chambers & Silver 2004). The latter study did, however, make conservative assumptions regarding the extent to which CO_2_ may stimulate tropical forest productivity (a 25% stimulation of *N*_P_ for an increase in [CO_2_] from approx. 270 to 700 μmol mol^−1^), so their result is not that surprising. In any case, it is not at all clear that experiments exposing plants to large-step changes in [CO_2_], such as in typical CO_2_ enrichment experiments, provide an adequate analogue for probable growth responses when [CO_2_] is gradually increasing, such as is presently the case. For example, seedlings can only typically adjust their ratios of root to shoot by a factor of less than 0.2 in response to a doubling of [CO_2_] (Curtis & Wang 1998), but for mature tropical forests an approximately threefold variation in the ratios of root to shoot exists, probably due to variations in nutrient availability (Maycock & Congdon 2000; Powers *et al*. 2005). Thus, although it may be the case that nutrients, especially P, are becoming relatively more limiting for tropical forest growth as *N*_P_ and [CO_2_] continue to increase (Chambers & Silver 2004), it would also be remarkable if tropical forest trees were not gradually increasing the proportion of biomass allocated below ground to facilitate relatively greater rates of nutrient acquisition. Moreover, numerous mechanisms exist that allow extra phosphorus to be taken up from the soil solution to support increased growth in response to higher [CO_2_] (Lloyd *et al*. 2001). Plants, it seems, have ready access to what are often considered ‘unavailable’ phosphorus pools (Parfitt 1979; Johnson & Loeppert 2006), much of which should be available to support slowly increasing [CO_2_]-mediated increases in growth (cf. Chambers & Silver 2004).

It has also been argued that because tropical tree carbohydrate concentrations, [CH_2_O], are ‘generally high’, [CH_2_O] must already be in excess with increasing [CO_2_] unable to further stimulate plant growth (Körner 2003; Wright 2005). That plants growing under elevated [CO_2_] often have higher [CH_2_O] has also been taken as additional evidence for this idea (Körner 2003). Nevertheless, recent advances in our understanding of the signalling of growth responses in plants, in particular interactions between sugar and plant hormone signalling with nutrients and other growth limitations (Rolland *et al*. 2006), show that higher [CH_2_O] is, in fact, usually associated with a stimulation of sink activity (i.e. faster rates of growth). Thus, increases in plant tissue [CH_2_O] with higher [CO_2_] do not mean that plant growth is not ‘carbon’ limited (Masle *et al*. 1990) and sugar signalling provides a simple explanation for why increased [CO_2_] usually causing increases in both [CH_2_O] and *N*_P_. For a sugar sensing mechanism to work, it must be the case that, on average, any change in [CH_2_O] gives rise to a less than proportional change in *N*_P_. Conceptually this bears a strong resemblance to the general economic theory of Keynes (1936)—in particular the notion that in order for an economic system in which savings occur to be able to operate, it is necessary for the long-term ratio of expenditure to income to always be less than unity. In that respect, the need for plants to maintain considerable CH_2_O reserves as insurance against drought, defoliation by pests or unexpected shading by competitors should not be discounted.

It is seedlings, saplings and trees growing under shaded conditions that tend to be the most carbon limited (Wright 2005) and plants adapted to and/or growing in shade are the most responsive to elevated [CO_2_] (Curtis & Wang 1998; Kerstiens 2001). A strong sensitivity of tropical forest seedling growth to elevated [CO_2_] has also been demonstrated (Würth *et al*. 1998), consistent with carbohydrate storage enhancing shade and stress tolerances for tropical forest seedlings (Myers & Kitajima 2007).

It thus seems likely to us that the currently observed accelerating dynamics of Amazon forests can reasonably be attributed to increases in [CO_2_], mediated at the seedling stage, although other factors such as changing light levels may, of course, also be involved (Wright 2005). Observations of increased growth being followed by increased mortality rates (Lewis *et al*. 2004) are also both conceptually and quantitatively consistent with ecosystem-level stimulations of *G*_P_ and *N*_P_ associated with slowly increasing [CO_2_] (Lloyd & Farquhar 1996). Although this provides a plausible mechanism for the observed accelerating dynamics of tropical forests, there must be a limit to the maximum size that any forest can attain. Our inability to understand the basis of variations in aboveground carbon stocks for all but the driest Amazon forests (Saatchi *et al*. 2007) currently limits our understanding of how long any sequestration is likely to continue.

## Figures and Tables

**Figure 1:**
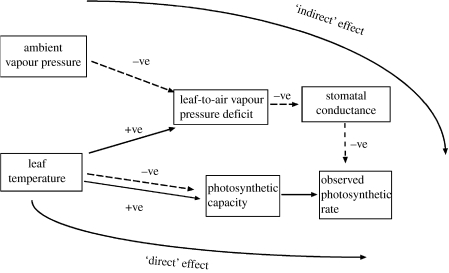
Schematic showing ‘direct’ and ‘indirect’ effects of temperature on leaf photosynthetic metabolism.

**Figure 2:**
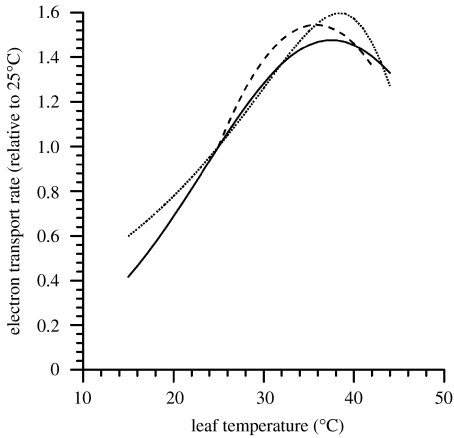
The temperature sensitivity of electron transport as deduced from the studies of June *et al*. (2004) with soya bean, and Tribuzy (2005) and Mercado *et al*. (2006) for Amazon forest trees. Dashed line, Tribuzy; dotted line, Mercado; solid line, June.

**Figure 3:**
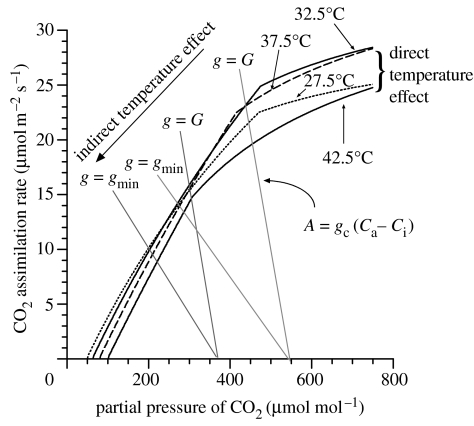
Modelled response of photosynthesis and stomatal conductance *g* to the intercellular/chloroplastic partial pressure of CO_2_ for various leaf temperatures and with operating points at the modelled maximum stomatal conductance *G* of 0.6 mol m^−2^ s^−1^, along with that associated with the maximum leaf-to-air vapour pressure observed within the model for 2000 and 2040 with [CO_2_]=380 and 550 μmol mol^−1^, respectively (denoted *g*_min_). The lines intersecting the *x*-axis represent the ‘stomatal supply’ functions (Farquhar & Sharkey 1982) for *g*=*G* and *g*=*g*_min_ at [CO_2_]=380 μmol ml^−1^ and [CO_2_]=550 μmol ml^−1^.

**Table 1:** Model estimates for annual net CO_2_ assimilation, maximum leaf temperature and maximum leaf-to-air vapour pressure difference for a leaf growing at the top of the canopy near Manaus for 2000 and 2040 in the absence of soil water deficits. (For 2040, simulations have been done both with the assumed [CO_2_] for 2000 (380 μmol mol^−1^) and for a more likely [CO_2_] around that time of 550 μmol mol^−1^. Two model assumptions for stomatal conductance *g* have been invoked: first, with *g*≡0.6 mol m^−2^ s^−1^ (minimal stomatal limitation); and secondly, and more realistically, with *g* responding to variations in leaf-to-air vapour pressure deficit and linking with leaf biochemistry according to equation (E5). The most likely values are shown in italics.)

		2000 climate	2040 climate	
model run	output parameter	[CO_2_]=380 μmol mol^−1^	[CO_2_]=380 μmol mol^−1^	[CO_2_]=550 μmol mol^−1^
*g*≡0.6 mol m^−2^ s^−1^	annual net CO_2_ assimilation (mol C m^−2^ a^−1^)	287.6	294.8	379.3
	maximum leaf temperature (°C)	34.2	35.8	35.8
	maximum leaf-to-air vapour pressure difference (mmol mol^−1^)	20.8	26.8	26.8
interactive *g* from equation (E5)	annual net CO_2_ assimilation (mol C m^−2^ a^−1^)	*207.4*	188.7	*271.1*
	maximum leaf temperature (°C)	*37.9*	39.7	*39.7*
	maximum leaf-to-air vapour pressure difference (mmol mol^−1^)	*33.0*	40.7	*40.8*

## Supplementary Material

Photosynthesis model description and note on increasing temperature responses Description of photosynthesis model with a note on stomatal responses to [CO_2_] and the response of tropical forest respiration rates to increasing temperatures.
