# Supplementary material for: Effects of rising temperatures and [CO2] on the physiology of tropical forest trees
Source: Philos Trans R Soc Lond B Biol Sci. 2008 Feb 11;363(1498):1811–7. doi: 10.1098/rstb.2007.0032 (PMC2374913; doi:10.1098/rstb.2007.0032)
Supplement: Supplementary file 1 [file rstb20070032s03.pdf]

ELECTRONIC SUPPLEMENTARY MATERIAL

A: DESCRIPTION OF PHOTOSYNTHESIS MODEL

The stomatal model of Jarvis & Davies (1998) can be written (Buckley *et al.* 2003) as

$$g = \frac{G(A_m - A)}{1 + sD(A_m - A)} \quad , \qquad \text{(E1)}$$

where g is the stomatal conductance to water vapour diffusion, A is the observed net CO_2 assimilation rate, A_{max} is the value of A at saturating intercellular CO_2 concentration, c_i, and G is the maximum possible stomatal conductance which occurs when $D = 0$ and $A \rightarrow 0$, and s is a constant describing the response of g to changes in D, these assumed to be mediated via a direct sensing of the leaf evaporation rate (Mott & Parkhurst 1991). As discussed by Buckley *et al.* (2003) some sort of surrogate measure of guard cell [ATP], τ, may infact be more appropriate than $(A_m - A)$ as a measure of how much faster CO_2 could be fixed if stomata did not limit its supply.

As shown by Farquhar & Wong (1984) and Buckley *et al.* (2003) τ may be modelled as taking on two different values: τ_c which applies when the ribulose bisphosphate (RuBP) saturated rate of carboxylation, W_c, is greater than the rate which can be sustained by the current rate of electron transport, W with the alternative value, τ_j applying when $W_j < W_c$. As written for equations (A22) to (A24) in Buckley *et al.* (2003)

$$\tau_c = a_t - \kappa \frac{W_c}{W_j} \quad , \qquad \text{(E2)}$$

$$\tau_j = \frac{(a_t - \kappa)\left(\frac{V_r}{V_{max}} - 1\right)}{\left[\frac{W_c}{W_j}\right]\frac{V_r}{V_{max}} - 1} \quad , \qquad \text{(E3)}$$

and

$$\tau \equiv \tau_o + \tau_c \quad \text{if} \quad W_c > W_j \quad ,$$

$$\tau \equiv \tau_o + \tau_j \quad \text{else} \quad . \qquad \text{(E4)}$$

In equations (E2), (E3) and E4, a_t represents the total concentration of adenylates in the chloroplast (equal to τ + [ADP]), κ is the concentration of photophosphorylation sites, V_r is the CO_2 and Rubisco saturated potential rate of carboxylation (i.e. the carboxylation rate that would occur if carboxylation were limited by the potential RuBP pool size only), V_{max} is the rate of carboxylation when limited by

Rubisco activity only (i.e. saturated with both CO_2 and RuBP) and τ_o represents a basal ATP level provided by other processes such as mitochondrial respiration.

We first rewrite equation (E1) in terms of τ for the RuBP saturated case as

$$g = \frac{G\,\tau_c / (\tau_o + a_t)}{1 + sD\tau_c / (\tau_o + a_t)} \quad , \qquad \text{(E5)}$$

where the scaling ensures that $g = G$ when the guard cell [ATP] supply is at its maximum possible value. Noting also that one can write (Buckley *et al.* 2003)

$$g = \frac{(0.23 + 1.37\omega)A}{c_a - p_i / p_t} \quad . \qquad \text{(E6)}$$

with ω being the ratio of the total and stomatal conductances to water vapour, c_a the ambient concentration of CO_2 and with p_i and p_t being the intercellular CO_2 partial pressure and the total ambient pressure respectively, combining equations (E2),(E4),(E5) and (E6), we obtain

$$\frac{(0.23 + 1.37\omega)A}{c_a - p_i / p_t} = \frac{G\tau_c}{\tau_o + a_t + sD\tau_c} \quad . \qquad \text{(E7)}$$

We then define A and τ_c in terms of their underlying biochemistry. As shown by Farquhar *et al.* (1980);

$$A = \left(1 - \frac{\Gamma^*}{p_i}\right) \cdot \min\{W_c, W_j\} - R_d \quad , \qquad \text{(E8)}$$

where Γ^* is the photorespiratory compensation point and with W_c and W_j expressed as

$$W_c = \frac{V_{max} p_i}{p_i + K_c(1 + pO_2 / K_o)} \quad , \qquad \text{(E9)}$$

and

$$W_j = \frac{J p_i}{4(p_i + 2\Gamma^*)} \quad , \qquad \text{(E10)}$$

Combining equations (E2), (E4), (E5), (E7), (E8) and (E9), we obtain for the case where $W_c < W_j$

$$\frac{(0.23 + 1.37\omega)V_{max}(p_i - \Gamma^*)}{[p_i + K_c(1 + pO_2 / K_o)](c_a - p_i / p_t)} = \frac{G\left(\tau_o + a_t - \kappa \dfrac{4V_{max}(p_i + 2\Gamma^*)}{J[p_i + K_c(1 + pO_2 / K_o)]}\right)}{\tau_o + a_t + sD\left(\tau_o + a_t - \kappa \dfrac{4V_{max}(p_i + 2\Gamma^*)}{J[p_i + K_c(1 + pO_2 / K_o)]}\right)} \quad . \qquad \text{(E11)}$$

For which it is possible to solve numerically for p_i and hence stomatal conductance, g using the approach outlined in Appendix 3 of Buckley *et al.* (2003). Note that in equation (E11) we have ignored the respiratory term of equation (E8) on the basis that, especially at high leaf temperatures, foliar

respiration is substantially inhibited in the light (Atkin *et al.* 2000). Likewise for the case where $W_j < W_c$ we write, also ignoring the R_d term

$$\frac{(0.23+1.37\omega)J(p_i-\Gamma^*)}{4(p_i+2\Gamma^*)(c_a-p_i/p_t)}=\frac{G\left\{\tau_o+a_t-\kappa\left(\dfrac{V_r}{V_{max}}-1\right)\Big/\left(\left[\dfrac{W_c}{W_j}\right]\dfrac{V_r}{V_{max}}-1\right)\right\}}{\tau_o+a_t+sD\left\{\tau_o+a_t-\kappa\left(\dfrac{V_r}{V_{max}}-1\right)\Big/\left(\left[\dfrac{W_c}{W_j}\right]\dfrac{V_r}{V_{max}}-1\right)\right\}}\quad . \qquad \text{(E12)}$$

which can also be solved numerically.

B. MODEL PHOTOSYNTHETIC PARAMETERS AND THEIR TEMPERATURE SENSITIVITIES

Based on the work of Domingues *et al.* (2004) we take a V_{max} at 25 °C, $V_{m(25)}$, of 80 μmol m^{-2} s^{-1} with the maximum rate of electron transport at 25 °C taken as 1.9$V_{m(25)}$. The temperature sensitivity of V_{max} is paramaterised as in Bernacchi *et al.* (2003) but using the kinetic constants of von Caemmerer *et al.* (1994) calculated on the assumption that the leaf internal conductance to the diffusion to CO_2 is infinite, *viz* K_c = 40.4 Pa and K_o = 24.8 x 10^3Pa. The temperature sensitivity of electron transport is as in June *et al.* (2004) with the dependence of the electron transport rate, J upon incoming irradiance (I) being described as the hyperbolic minimum of the J_{max} and the product of I and F where F is the product of leaf absorbtivity to PAR and the effective quantum yield (Farquhar & Wong 1984).

As in Buckley *et al.* (2003) we take $\kappa = 2.5\ |V_{m(25)}|$ mmol sites m^{-2}, $a_t = 12.6\ |V_{m(25)}|$ mmol A*x*P m^{-2} where the $|V_{m(25)}|$ indicates a numerical value only . i.e. = $V_{m(25)}$ /(μmol m^{-2} s^{-1}), and with τ_o set to 1.6 mmol ATP m^{-2}. The ratio $V_r/\ V_{max}$ was taken as 2.27 (Farquhar & Wong 1984) and assumed to be independent of temperature. Based on observed stomatal responses to D as observed for Amazon forest from eddy covariance data (Mercado 2007) we took G as 0.6 mol m^{-2} s^{-1} with s = 0.122 mol^{-1} mol.

One additional feature of our model is that it takes into account the observation that although the rate of electron transport through photosystem II may (reversibly) decline at leaf temperatures, T_L, above that optimal for electron transport, T_{opt} (June *et al.* 2004); this is also associated with an increase in the cyclic flow of electrons around photosystem I which probably serves as an important mechanism for the protection of both PS II and lipid membranes under high temperature conditions (Sharkey & Schrader 2006), as well as the maintenance of high ATP levels at supraoptimal T_L (Schrader *et al.* 2004). Thus is the simulations here, we simply set τ_j equal to the τ_j calculated to occur at T_{opt} for all $T_L > T_{opt}$. This is an important feature of the model which still requires experimental verification in terms of

stomatal responses to temperatures that are supraoptimal in terms of whole chain electron transport itself.

C. DRIVING VARIABLES AND THE LEAF ENERGY BUDGET

The model, which includes a simple energy balance as described in Lloyd *et al.* (1995), is run for a single sun exposed leaf at the top of the canopy and on an hourly basis using modelled values for air temperature, absolute humidity, wind-speed and incoming shortwave radiation in for both 2000 and 2040. Driving data for the hourly simulations in 2000 came from an updated version of New *et al.* (2000). For 2040, estimates were obtained as the difference between Hadley Centre GCM values for 2040 and 2000 added to the New *et al.* (2002) climatology values for 2000. In both cases hourly values were obtained using the climate generator which is part of the IMOGEN program (Huntingford *et al.* 2004). Boundary layer conductances and leaf energy budgets, also allowing for forced convection, were estimated as described in the Appendix of Ball *et al.* (1994) with an average leaf area for the Manaus tower site taken as 21 cm^2 (S. Patiño *et al.* unpublished data). The wind speed taken at the top of the canopy (where our theoretical leaf resides) was taken directly from the IMOGEN output.

D. A NOTE ON STOMATAL RESPONSES TO CO_2

Although not explicitly included in our model, equation (2) gives rise to stomatal responses to ambient [CO_2] through the τ term in equation (E2). This is because [ATP] decline as [CO_2] increase. Nevertheless, the response in the model is complex, with stomata tending to be *relatively* less responsive to [CO_2] at high light and at high D (Buckley *et al.* 2003). The degree of stomatal closure in response to an increase in [CO_2] from 380 μmol mol^{-1} to 550 μmol mol^{-1} is thus quite small in our simulations. Clarke (2004) have, however, suggested that an increase in leaf temperatures associated with such stomatal closure may be critical in reducing tropical tree photosynthesis, perhaps even pushing some trees beyond their thermal limits, this being akin to the notion of "stomatal suicide" (Randall *et al.* 1996). We have thus tested the potential likelihood of this effect by reducing g_{max} by 25% (i.e. to 0.48 mol m^{-2} s^{-1}) and rerunning the fully interactive 2040 scenario with [CO_2] = 550 μmol mol^{-1}. This gives rise to a substantial reduction in the simulated Gross Primary Productivity, G_P (reduced from 271.1 to 232.9 mol C m^{-2} a^{-1}) due to substantially lower p_i, but only marginal increases in the maximum simulated leaf temperature and leaf-to-air vapour pressure deficit (from 39.7 to 40.4 °C and from 40.7 to 43.7 mmol mol^{-1} respectively). Although photosynthetic rates are substantially reduced by imposed stomatal closure in response to higher [CO_2], the simulated G_P for 2040 at an ambient [CO_2] of 550 μmol mol^{-1}

is still substantially higher than for 2000 for which the ambient $[CO_2]$ = 380 μmol mol^{-1}. According to these simulations then, there is no reason to assume that any stomatal closure at higher $[CO_2]$ should push tropical tree leaves dangerously close to their thermal limit or reduce their photosynthetic productivity below what is currently the case.

E. RESPIRATION, TEMPERATURE AND TROPICAL FOREST PRODUCTIVITY

Feeley *et al.* (2007) reported that, especially for their Pasoh site, a significant decline in community-level relative growth rates (RGR) of around 50% (for trees > 10 cm diameter at breast height) may have been attributable to increased respiration rates associated with an increase in minimum daily air temperatures of at most 0.7 °C over a 14 year period. One interesting question then is what the temperature sensitivity of respiration would have to be for this hypothesis to hold.

This can be simply calculated by first modifying equation (1) in the main text,

$$N_P = G_P(1 - \phi_o - \phi_m) \quad , \qquad \text{(E13)}$$

where ϕ_o represents the loss of carbon associated with the conversion of photosynthate to organic matter (typically around 0.2 and insensitive to temperature – see for example Lloyd & Farquhar 1996) and ϕ_m quantifies the loss of carbon through "maintenance" respiratory processes, also expressed as a fraction of G_P. Taking a recent estimate for ϕ_m of 0.45 (Malhi *et al.* 2008) and with ϕ_o as 0.2 (and so with φ in equation (1) of the main text equal to 0.65), this means that ϕ_m would have to increase from 0.45 to 0.625 (i.e. by ~ 39%) in order for N_P to decline by about 50%, suggesting relative sensitivity of maintenance respiration to temperature, B, of approximately 0.39/0.70 ~ 0.56 °C^{-1}. From such a calculation we can easily estimate Q_{10} as exp[10B] for which we obtain a Q_{10} for plant respiration of considerably more than 30.

Such a calculation is based on the assumption that the decline in stem productivity observed is proportionally the same throughout the entire plant. It may be, however, that new stem and structural root growth represent only the carbohydrate "leftovers" once carbohydrate for new leaf and fine root production have been utilised (Lloyd & Farquhar 1996). In which case, the required increase in ϕ_m would be considerably less. But even with only about 30% of new growth going into boles and structural roots, but with new growth associated with new leaves, branches and fine roots (which generally constitute about 70% of N_P (Malhi *et al.* 2008) conserved, then the decrease in overall N_P would be only ca 15% with a temperature sensitivity for ϕ_m of ~ 0.17 °C^{-1}. This leads to a Q_{10} of greater than 5 which still seems too high. Nevertheless, if that were to actually be the case, then it is worth pointing out that just looking at stem growth rates must also be considered as giving a greatly amplified

view of any changes in overall tree growth rates with time. And by corollary this also applying to any observed increases such as reported in Baker *et al.* (2004).

We further note that is by no means clear that long-term temperature effects will be of the same magnitude as interannual variations (Atkin *et al.* 2005), but even if so, for the projected increase in air temperatures between 2000 and 2040 being about 1.5 °C, then with a Q_{10} = 2.3 for Amazon forest respiration (Meir *et al.* 2001) and with no acclimation (and using the parameters above) ϕ_m would be expected to increase only by about 14% from 0.45 to around 0.51; i.e. φ in equation (1) of the main text would increase from 0.65 to 0.71. Thus, even taking a worse case scenario by allowing for a 25% reduction in stomatal conductances and no acclimation of respiration to increasing temperatures at all by 2040, then N_P would only decline by about 7% with the most probable value almost most certainly being much less than this and more likely a significant increase. For example, at the other extreme (assuming no stomatal closure and allowing for full acclimation of plant respiration) then N_P would be modelled to increase by about 33% between 2000 and 2040 and with stem growth rates actually doubling over that 40 year period if it were to be the case that all increased N_P is channelled towards boles and fine roots (as discussed above).

F. REFERENCES

Atkin, O.K, Evans, J., Ball, M.C., Lambers, H. & Pons, T.L. 2000 Leaf respiration of snow gum in the light and dark. Interactions between temperature and irradiance. *Plant Physiol.* **122,** 915-924.

Atkin, O. K., Bruhn, D., Hurry, V. M. & Tjoelker, M. G. 2005. The hot and the cold: unravelling the variable response of plant respiration to temperature. *Funct. Plant Biol.* **32,** 87-105.

Baker, T.R. *at al.* 2004 Increasing biomass in Amazonian forest plots. *Phil. Trans.Royal Soc. Lond. Ser. B* **359,** 353-365.

Ball, M. C., Cowan, I. R. & Farquhar, G. D. 1984 Maintenance of leaf temperature and the optimisation of carbon gain in relation to water loss in a tropical mangrove forest. *Aust. J. Plant Physiol.* **15,** 263-276.

Bernacchi, C. J., Singsaas, E. L. Pimental, C., Portis Jr., A. R. & Long, S. P. 2001 Improved temperature response functions for models of Rubisco-limited photosynthesis. *Plant Cell & Environ.* **24,** 253 -259.

Buckley, T. N., Mott, K. A. & Farquhar, G. D. 2003 A hydromechanical and biochemical model of stomatal conductance. *Plant Cell & Environ.* **26,** 1767 -1785.

Clark, D. A. 2004 Sources or sinks? The responses of tropical forests to current and future climate and atmospheric composition. *Phil. Trans. R. Soc. Lond.* B **359,** 477-491.

Domingues, T. F., Berry, J. A. Martinelli, L.A., Ometto, J.P. and Ehleringer, J.R. 2005 Paramaterisation of canopy gas exchange and leaf-level gas exchange for an eastern Amazonian tropical rain forest (Tapajós National Forest, Pará, Brazil) *Earth Interactions* **9,** 17.

Farquhar, G. D. & Wong, S. C. 1984, An empirical model of stomatal conductance. *Aust. J. Plant Physiol.* **11,** 191-210.

Farquhar, G. D. von Caemmerer, S. & Berry, J. A. 1980 A biochemical model of the photosynthetic assimilation in C_3 species. *Planta* **149,** 78-90.

Feeley, K. J., Wright, S. J., Supardi, N. N. N., Kassim, A. R., Davies, S. J. 2007. Decelerating growth in tropical forest trees. *Ecol. Lett.* **10,** 461-469.

Huntingford, C., Harris, P. P., Gedney, N., Cox, P. M., Betts, R. A., Marengo, J. A. & Gash, J. H. C. 2004. Using a GCM analogue model to investigate the potential for Amazonian forest dieback. *Theor. Appl. Climatol.* **78,** 177-185.

Jarvis, A. J. & Davies, W. J. (1998) The coupled response of stomatal conductance to photosynthesis and transpiration. *J. Exp. Bot.* **49,** 399-406.

June, T., Evans, J. R. & Farquhar, G. D. 2004, A simple new equation for the reversible temperature dependence of photosynthetic electron transport: a study on soybean leaf. *Funct. Plant Biol.* **31,** 275-283.

Lloyd, J. & Farquhar, G.D. 1996 The CO_2 dependence of photosynthesis, plant growth responses to elevated atmospheric CO_2 concentrations and their interaction with plant nutrient status. *Funct. Ecol.* **10,** 4-32.

Lloyd, J., Grace, J., Miranda, A.C., Meir, P., Wong, S-C., Miranda, H.-S., Wright, I.R., Gash, J.H.C. & MacIntyre, J.A. 1995 A simple calibrated model of Amazon rainforest productivity based of leaf biochemical properties. *Plant Cell Environ.* **18,** 1129-1145.

Malhi, Y. *et al.* 2008 Comprehensive assessment of carbon productivity, allocation and storage in three Amazonian forests. *Global Change Biology* (submitted).

Meir, P., Grace, J. & Miranda, A.C. 2001. Lead respiration in two tropical rainforests: constraints on physiology by phosphorus, nitrogen and temperature. *Funct. Ecol.* **15,** 378-387.

Mercado, L.M.M. 2007 *Modelling Rainforest Canopy Photosynthesis across the Amazon Basin.* Ph.D thesis, Free University of Amsterdam.

Mott, K. A. & Parkhurst, D. F. 1991 Stomatal response to humidity in air and helox. *Plant Cell & Environ.* **14,** 50.9-515.

New, M., Lister, M., Hulme, M. & Makin, I. 2000 A high-resolution data set of surface climate over global land areas. *Climate Res.* **21,** 1-25.

Randall D.A., Dazlich D.A., Zhang C., Denning A.S., Sellers P.J., Tucker C.J., Bounoua L., Berry J.A., Collatz G.J., Field C.B., Los S.O., Justice C.O. & Fung I. 1996 A revised land surface parameterization (SiB2) for GCMs. Part III: the greening of the Colorado State University general circulation model. *J. Climate*, **9**, 738-763.

Schrader, S. M., Wise, R. R., Wacholtz, W. F., Ort, D. R. & Sharkey, T. D. 2004 Thylakoid membrane response to moderately high leaf temperature in Pima cotton. *Plant Cell & Environ.* **27,** 725-735.

Sharkey, T. D. & Schrader, S. M. 2006 High temperature sense. In *Physiology and Molecular Biology of Stress Tolerance in Plants* (eds. K. V. M. Rao, A. S. Raghavendra & K. J. Reddy) pp101-129. Springer, Dordrecht, The Netherlands.

von Caemmerer, S., Evans, J.R., Hudson, G.S. & Andrews, T.J. 1994 The kinetics of ribulose-1,5-bisphosphate carboxylases/oxygenase *in vivo* inferred from measurements of photosynthesis in leaves of transgenic tobacco. *Planta* **196,** 88-97.
